# Identification of a *Spotted Leaf Sheath* Gene Involved in Early Senescence and Defense Response in Rice

**DOI:** 10.3389/fpls.2018.01274

**Published:** 2018-09-05

**Authors:** Dongryung Lee, Gileung Lee, Backki Kim, Su Jang, Yunjoo Lee, Yoye Yu, Jeonghwan Seo, Seongbeom Kim, Yong-Hwan Lee, Joohyun Lee, Sunghan Kim, Hee-Jong Koh

**Affiliations:** ^1^Department of Plant Science, Plant Genomics and Breeding Institute, and Research Institute of Agriculture and Life Science, Seoul National University, Seoul, South Korea; ^2^Department of Agricultural Biotechnology, Center for Fungal Genetic Resources, and Center for Fungal Pathogenesis, Seoul National University, Seoul, South Korea; ^3^Department of Applied Bioscience, Graduate School of Konkuk University, Seoul, South Korea; ^4^Department of Biological Science, Sookmyung Women's University, Seoul, South Korea

**Keywords:** lesion mimic mutant (LMM), leaf sheath, early senescence, reactive oxygen species (ROS), blast resistance, Mitogen-Activated Protein Kinase Kinase Kinase (MAPKKK)

## Abstract

Lesion mimic mutants (LMMs) commonly exhibit spontaneous cell death similar to the hypersensitive defense response that occurs in plants in response to pathogen infection. Several lesion mimic mutants have been isolated and characterized, but their molecular mechanisms remain largely unknown. Here, a *spotted leaf sheath* (*sles*) mutant derived from *japonica* cultivar Koshihikari is described. The *sles* phenotype differed from that of other LMMs in that lesion mimic spots were observed on the leaf sheath rather than on leaves. The *sles* mutant displayed early senescence, as shown, by color loss in the mesophyll cells, a decrease in chlorophyll content, and upregulation of chlorophyll degradation-related and senescence-associated genes. ROS content was also elevated, corresponding to increased expression of genes encoding ROS-generating enzymes. Pathogenesis-related genes were also activated and showed improved resistance to pathogen infection on the leaf sheath. Genetic analysis revealed that the mutant phenotype was controlled by a single recessive nuclear gene. Genetic mapping and sequence analysis showed that a single nucleotide substitution in the sixth exon of *LOC_Os07g25680* was responsible for the *sles* mutant phenotype and this was confirmed by T-DNA insertion line. Taken together, our results revealed that *SLES* was associated with the formation of lesion mimic spots on the leaf sheath resulting early senescence and defense responses. Further examination of *SLES* will facilitate a better understanding of the molecular mechanisms involved in ROS homeostasis and may also provide opportunities to improve pathogen resistance in rice.

## Introduction

Leaf senescence, the final stage of leaf development, is primarily governed by leaf age. However, leaf senescence is also influenced by various internal and environmental signals that are integrated with the age information (Lim et al., 2007). Lesion mimic mutants (LMMs) are often associated with early leaf senescence. An *spl5* mutant continuously developed small reddish-brown necrotic lesions on leaves, leading to early senescence (Chen et al., 2012). An *lmes1* mutant also exhibited early senescence, with tiny brown spots developing initially at the leaf tip and spreading to the entire leaf surface (Li et al., 2014). LMMs can be divided into two classes according to the mechanisms involved in controlling cell death: (1) initiation mutants and (2) feedback or propagation mutants (Lorrain et al., 2003). Initiation mutants, such as *acd5, cpn1*, and *cpr5*, form localized necrotic spots of determinate size whereas formation rate and lesion extent are not controlled in propagation mutants, such as *acd2, lsd1*, and *svn1* (Dietrich et al., 1997; Boch et al., 1998; Lin and De, 1999; Greenberg et al., 2000; Jambunathan et al., 2001; Mach et al., 2001).

The necrotic spots formed in LMMs resemble those formed during the pathogen infection-induced hypersensitive response (HR). Reactive oxygen species (ROS) are thought to prime the orchestration of the HR (Zurbriggen et al., 2010). During HR, rapid production of ROS, such as hydrogen peroxide (H_2_O_2_), superoxide (O${}_{2}^{-}$), is stimulated in mitochondria and chloroplast as well as in the cytoplasmic level (Zurbriggen et al., 2010). Plants use two types of defense mechanisms to combat oxidative stress. These mechanisms involve non-enzymatic antioxidants such as ascorbate and glutathione, or enzymatic antioxidants such as catalase, superoxide dismutase, and ascorbate peroxidase (Navabpour et al., 2003). Either over-accumulation of ROS or the failure of these oxidative stress defense mechanisms will result in cell death. HR is an important resistance mechanism that prevents pathogen spread to adjacent cells by inducing cell death in infected regions (Lam et al., 2001). HR is usually accompanied by the activation of pathogenesis-related (*PR*) genes. Expression of *PR1a* and *PR1b*, which encodes acidic and basic proteins, respectively, was induced upon infection with rice blast fungus (Agrawal et al., 2000a,b). Expression of *PR5* family genes, which encodes thaumatin-like proteins, was induced in plants in response to infection by plant pathogens, elicitors, stresses, and developmental signals (Bryngelsson and Green, 1989). Proteins of the PR10 family were involved in multiple anti-pathogen processes, and are generally localized in the intracellular spaces, in contrast to the extracellular nature of most PR proteins (Jwa et al., 2001).

LMM genes encode wide range of functional protein types, such as heat stress transcription factor (Yamanouchi et al., 2002), U-Box/Armadillo repeat protein (Zeng et al., 2004), zinc finger proteins (Wang et al., 2005), membrane-associated proteins (Noutoshi et al., 2006), ion channel family member (Mosher et al., 2010), clathrin-associated adaptor protein (Qiao et al., 2010), and splicing factor 3b subunit 3 (Chen et al., 2012), indicating the involvement of complex and diverse molecular mechanisms in lesion mimic spot formation. Recently, Wang et al. (2015) showed that Mitogen-Activated Protein Kinase Kinase Kinase (MAPKKK) was also involved in the formation of lesion mimic spots. Several studies revealed that MAPKKK plays an important role in regulating a range of biological processes, such as ethylene signaling (Kieber et al., 1993), plant cytokinesis (Krysan et al., 2002), innate immunity (Asai et al., 2002), defense responses (Suarez-Rodriguez et al., 2007), responses to various stresses (Gao and Xiang, 2008), stomatal development (Kim et al., 2012), and ABA signaling (Wang et al., 2015). However, the relationship between lesion mimic spot formation and MAPK cascades remains poorly understood.

In this study, a new lesion mimic mutant (*sles*) was identified. In contrast to other LMMs, which exhibited necrotic spots on leaves, lesion mimic spots in the *sles* mutant covered the leaf sheath resulting in early senescence. Fine-mapping and sequence analysis revealed that the *sles* locus encoded a kinase domain (KD) containing protein of the Raf MAPKKK family. Greenness and chlorophyll content were adversely affected in mesophyll cells in the *sles* mutant. Expression of genes encoding ROS-generating enzymes was induced in the *sles* mutant and ROS accumulation increased accordingly. Defense response genes were also activated and showed enhanced resistance to pathogen infection in *sles* mutant. These results are relevant to future research into the mechanisms involved in the formation of lesion mimic spots, ROS homeostasis, and resistance to diseases in plants.

## Materials and methods

### Plant material and growth conditions

The *sles* mutant was isolated through EMS treatment of the *japonica* cultivar Koshihikari. The *sles* mutant was crossed with Koshihikari (*japonica*) and Milyang 23 (M.23). The M.23 genetic background is similar to that of *indica*. For phenotypic characterization and genetic mapping, plants were grown by conventional culture at the Experimental Farm of Seoul National University, Suwon, Korea. F_2_ populations and parents were seeded in a plastic tunnel seedbed. Forty-day-old seedlings were then transplanted, one plant per hill, into a paddy field. The two-tailed Student *t*-test was used to compare the agronomic traits of *sles* mutant and wild type plants. 10 independent plants were measured to calculate the means values.

### Anatomical characterization

For light microscopic study, thin sections of 100-day-old wild-type penultimate leaf sheath and non-spotted and spotted regions of the *sles* mutant leaf sheath were cut using a sharp blade and observed using an Olympus CX31 dissecting microscope (Olympus, Japan) under white light. Photographs were taken using an Olympus eXcope T500 digital camera (Olympus, Japan). Three independent plants were used for the anatomical characterization.

### Chlorophyll and carotenoid content measurements

Chlorophyll (Chl) a, Chlb, and carotenoid (Car) contents were assessed in the penultimate leaf sheath from 100-day-old wild-type and *sles* mutant plants. Absorption values were measured as described by Arnon (1949) using UV/Vis spectrophotometer (Biochrom Libra S22, USA). The two-tailed Student *t*-test was used to compare the pigment intensity between *sles* mutant and wild type plants. Three biological replicates were used for the experiment.

### Histochemical characterization

For O${}_{2}^{-}$ determination, penultimate leaves and leaf sheath samples from 100-day old plants were vacuum-infiltrated (three cycles of 10 min) in 0.5 mg ml^−1^ nitro blue tetrazolium (NBT) in 10 mM potassium phosphate buffer (pH 7.8) for 16 h. For H_2_O_2_ detection, samples were vacuum-infiltrated (three cycles of 10 min) in 1 mg ml^−1^ 3,3′-diaminobenzidine (DAB) containing 10 mM MES (pH 6.5) for 16 h. Reactions were stopped by transferring tissue to 90% ethanol and incubating at 70°C until chlorophyll was completely removed. The cleared leaves and leaf sheaths were examined and photographed after a 2–4 h incubation period. Trypan blue staining was performed on fresh leaf and leaf sheath as previously described by Qiao et al. (2010). Samples were submerged in lactic acid-phenol-trypan blue solution (LPTB; 2.5 mg ml^−1^ trypan blue, 25% (w/v) lactic acid, 23% water-saturated phenol, and 25% glycerol in H_2_O) at 70°C, infiltrated by slow-release vacuum for 4 min, and then re-infiltrated. Samples in LPTB were heated in boiling water for 2 min and then cooled for 1.5 h before LPTB solution was replaced with visikol for destaining. The cleared leaves and leaf sheaths were examined and photographed after 3 d incubation period. All the experiments were performed in 10 biological replicates.

### Blast resistance evaluation

The *Magnaporthe oryzae* strain KJ201 was provided by Department of Agricultural Biotechnology, College of Agriculture and Life Sciences, Seoul National University, Seoul, Korea. The *sles* mutant and wild-type plants were grown in the greenhouse at 28/25°C, day/night, and were inoculated with KJ201 suspension into the leaf sheath of 50-day-old seedlings in a procedure described by Koga et al. (2004). The blast resistance evaluation was determined 2 days after inoculation by the invasive hyphae on inoculated leaf sheath.

### RNA isolation and real-time PCR

Total RNA was extracted from the penultimate leaf sheath of 100-day-old *sles* mutant and wild-type plants using Iso-Plus reagent (Takara Bio, Japan) according to the manufacturer's instructions. RNA was then treated with RNA-free DNase I (Promega, USA) to remove any remaining genomic DNA. DNase-treated RNA was reverse transcribed to first-strand cDNA using M-MLV reverse transcriptase (Promega, USA). Real-time PCR was performed using a CFX96 Real-time PCR detection system with SYBR Premix Ex Taq (Takara Bio, Japan). Primer3web (http://bioinfo.ut.ee/primer3/) was used to design primers that spanned an intron to enhance specific amplification of target fragments. Primers used for gene-specific PCR are listed in Supplementary Table 1. Data were analyzed using the comparative Ct method. The two-tailed Student *t*-test was used to compare the expression level between *sles* mutant and wild type plants.

### Genetic analysis and molecular mapping of *sles*

For genetic analysis, F_2_ populations were developed from two crosses: *sles* mutant × M.23 and *sles* mutant × Koshihikari. Bulked segregant analysis (BSA) was performed for preliminary genetic mapping using sequence-tagged site (STS) markers designed at the Crop Molecular Breeding Lab, Seoul National University (Chin et al., 2007). Two molecular markers flanking the primary candidate region were used to screen recombination events from 628 F_2_ individuals. To fine map *sles*, new STS markers between the two flanking markers were designed based on the sequence difference between *japonica* variety Nipponbare and the *indica* variety 93-11. Primers used for genetic mapping are listed in Supplementary Table 1.

### Sequence analysis of candidate genes

Gene prediction analysis was performed using the Gramene database (http://www.gramene.org) and *sles* candidate genes were analyzed further. The sequence of the AP005101 BAC clone was used to design 28 specific primers for sequence analysis of *sles* candidate genes. PCR-amplified products were purified using Inclone^TM^ Gel & PCR purification kit (Inclone Biotec, Republic of Korea), TA-cloned into the pGEM-T Easy Vector (Promega, USA), and transformed into *E. coli* strain DH5α for sequencing.

### Genotyping of T-DNA insertion mutants

Twenty dehulled T_1_ seeds were surfaced-sterilized, placed on 1/2 MS media containing 50 mg/ml hygromycin, and allowed to germinate in the dark at 37°C. To genotype T-DNA insertion lines, three primers were designed based on sequence information for T-DNA insertion positions available at RiceGE (http://signal.salk.edu/cgi-bin/RiceGE). Primers used for PCR are listed in Supplementary Table 1. PCR was used to test co-segregation between flanking sequences and the mutation phenotype.

### Amino acid sequence alignment and phylogenetic relationship

Homologs of SLES were identified in other species using search functions at the NCBI website. Multiple sequence alignments were conducted using Clustal X (http://www.clustal.org/) and edited with BOXSHADE (http://www.ch.embnet.org/software/BOX_form.html). NCBI web-based searches were used for conserved domain prediction of the SLES protein (https://www.ncbi.nlm.nih.gov/cdd).

## Results

### Phenotypic characterization of the *sles* mutant

**Figure 1:**
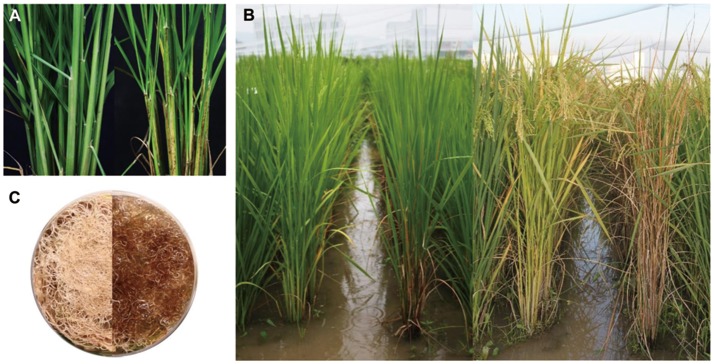
Morphological comparisons between wild-type and *sles* mutant plants. **(A)** Wild type (left) and *sles* mutant (right) at 60 day after germination. **(B)** 90-day-old and 120-day-old wild type (left) and *sles* mutant (right). **(C)** Root color in wild type (left) and *sles* mutant (right).

Wild-type and mutant plants were phenotypically and agronomically compared. The mutant exhibited lesion mimic spots on the leaf sheath (Figure 1A). Sparse spots appeared initially at the two-leaf stage and later expanded to cover the entire leaf sheath, resulting in earlier senescence than in wild type (Figure 1B). Lesion mimic spots were restricted to the leaf sheath and were not observed on leaves. Based on these observations, the mutant was designated *spotted leaf sheath* (*sles*). Lesion mimic spots also appeared on *sles* mutant roots (Figure 1C), initiating at the same growth stage as spot appearance on the leaf sheath.

Wild-type and *sles* mutant plants were significantly different with respect to agronomic traits (Table 1). Seed germination rate was substantially lower in the mutant compared to wild-type. Seedling vigor at the 3-leaf-seedling stage, as determined by shoot length, root length, fresh weight, and dry weight, was significantly reduced in the *sles* mutant compared to wild type. Leaf emergence was also slower in the *sles* mutant than in wild type. In the reproductive stage, plant height and number of tillers were significantly reduced in the *sles* mutant compared to wild type. Grains were smaller and thinner in the *sles* mutant than in wild-type. However, grain shape, which was determined by the grain length/width ratio, was similar between wild type and the *sles* mutant. Heading was delayed by a week in the *sles* mutant compared to wild type. Nevertheless, the *sles* mutant senesced more rapidly than wild type and *sles* leaves yellowed 4 weeks after heading while wild-type plants remained green (Figure 1B). Yield-related agronomic traits such as spikelet number per panicle, seed-setting rate, and 1,000-grain weight were all significantly adversely impacted compared to wild type.

**Table 1:** Agronomic traits of *sles* mutant and wild type plants.

**Traits**	**GR (%)**	**SL (cm)**	**RL (cm)**	**FW (mg)**	**DW (mg)**	**PH (cm)**	**TN (No.)**
WT	86.5 ± 9.2	16.2 ± 1.7	12.7 ± 1.8	123.6 ± 10.6	22.0 ± 2.1	113.1 ± 2.2	11.1 ± 2.5
*sles*	52.7 ± 1.8	13.8 ± 2.1	11.2 ± 1.5	91.4 ± 14.4	15.2 ± 1.5	97.3 ± 5.7	6.1 ± 1.7
Difference	^**^	^**^	^*^	^**^	^**^	^**^	^**^
**Traits**	**GL (mm)**	**GW (mm)**	**LWR**	**SN (No.)**	**SF (%)**	**KGW (g)**	
WT	0.71 ± 0.03	0.33 ± 0.01	2.2 ± 0.1	122.7 ± 18.5	90.7 ± 3.8	25.0 ± 0.7	
*sles*	0.67 ± 0.02	0.30 ± 0.01	2.2 ± 0.1	100.8 ± 24.3	69.4 ± 6.9	17.4 ± 1.9	
Difference	^**^	^**^	NS	^*^	^**^	^**^	

GR, germination rate; SL, shoot length; RL, root length; FW, fresh weight; DW, dry weight; PH, plant height; TN, tiller number; GL, grain length; GW, grain weight; LWR, GL/GW ratio; SN, number of spikelets per panicle; SF, spikelet fertility; KGW, 1000-grain weight; NS, not significant. 10 independent plants were measured to calculate the means value. Asterisks indicate statistical significance level according to Student's t-test:

** P < 0.01 and

* *P < 0.05*.

### Anatomical characterization of the *sles* mutant

**Figure 2:**
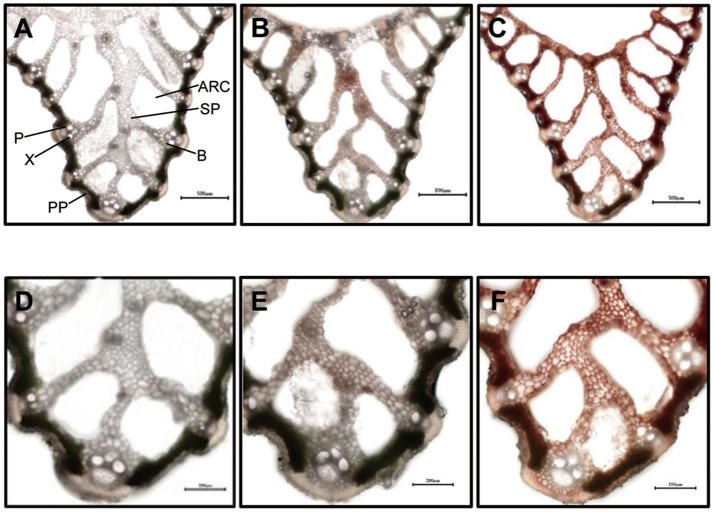
Light microscopic analysis of spotted and non-spotted leaf sheath from *sles* mutant and wild type plants. Transverse sections of penultimate leaf sheaths were observed under white light. **(D–F)** are magnified view of **(A–C)**, respectively. **(A,D)** are wild type leaf sheath sections. **(B,E)** are non-spotted, and **(C,F)** are spotted leaf sheath sections from the *sles* mutant. Indications in **(A)** are ARC, aerenchyma; B, bundle sheath; P, phloem; PP, palisade parenchyma; SP, spongy parenchyma; X, xylem.

Differences in mesophyll greenness level were apparent between wild type and *sles* mutant tissues (Figure 2). Palisade parenchyma of mesophyll cells were green and filled with chloroplast in leaf sheath sections from wild type and non-spotted regions close to spots in the *sles* mutant (Figures 2A,B,D,E). However, although the palisade parenchyma of mesophyll cells from non-spotted *sles* regions were green, dark brown areas were sometimes observed in the spongy parenchyma of the mesophyll cells (Figures 2B,E). In the spotted region of *sles* mutant leaf sheath, however, mesophyll cells were completely dark brown (Figures 2C,F), indicating their death.

### Leaf sheath chlorophyll and carotenoid content in the *sles* mutant

**Figure 3:**
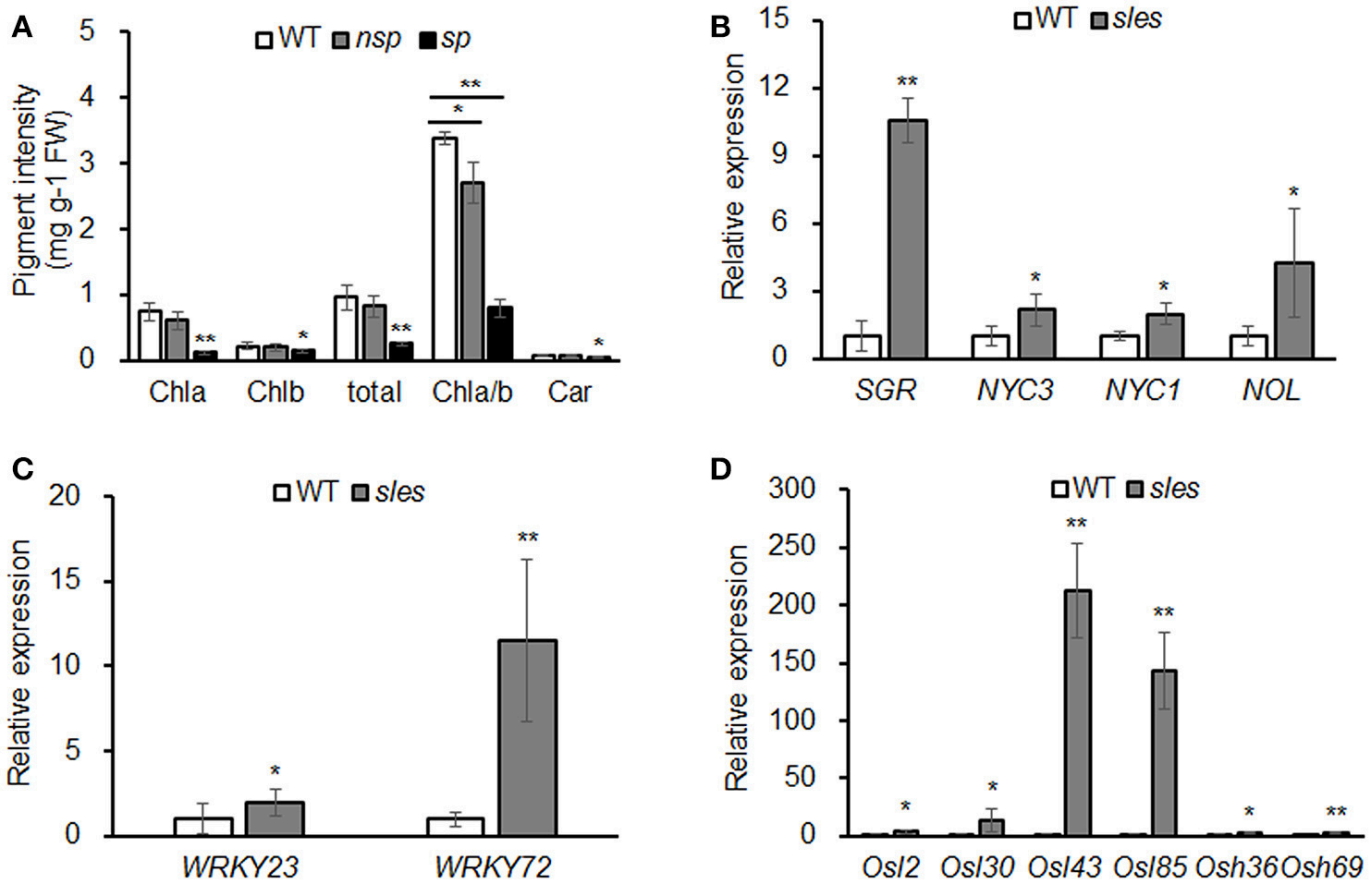
Early senescence in the *sles* mutant leaf sheath. **(A)** Abundance of major plant pigments in non-spotted (*nsp*) and spotted (*sp*) leaf sheaths from the *sles* mutant and wild-type (WT) leaf sheaths. **(B)** Expression of chlorophyll degradation-related genes. **(C)** Expression of senescence transcription factors. **(D)** Expression of senescence-associated genes. Real-time PCR (three biological replicates and three technical replicates) was performed with WT leaf sheath samples and *sles* leaf sheath samples from regions with legion mimic spots. Asterisks indicate the statistical significance levels according to Student's *t*-test: ^**^P < 0.01 and ^*^P < 0.05.

Contents of chlorophyll and carotenoid, the two most important pigments in rice, were compared between *sles* mutant and wild type (Figure 3A). Total chlorophyll content and Chla and Chlb levels were significantly lower in *sles* spotted regions than in wild type. The Chla/Chlb ratio was also significantly lower in the *sles* mutant than in wild type, indicating that Chla content had decreased to a greater extent than Chlb content in the *sles* mutant. The Chla/Chlb ratio was also significantly different between the non-spotted region of the *sles* mutant and the wild type, but there were no apparent differences in total chlorophyll content or Chla and Chlb levels. Carotenoid content was also significantly lower in the spotted region of the *sles* mutant leaf sheath than in wild type. Overall, the dark brown spotted regions of the *sles* mutant leaf sheath correlated with reductions in chlorophyll content.

### Senescence-related gene expression in the *sles* mutant

We examined the expression of chlorophyll degradation-related genes such as *STAY GREEN* (*SGR*), *NON-YELLOW COLORING* (*NYC1*), *NON-YELLOW COLORING3* (*NYC3*), and *NYC1-like* (*NOL*) (Kusaba et al., 2007; Park et al., 2007; Morita et al., 2009; Sato et al., 2009). Expression analysis showed that the chlorophyll degradation-related genes, particularly *SGR*, were dramatically upregulated in the *sles* mutant compared to wild type (Figure 3B). To confirm that senescence occurred in the leaf sheath of the *sles* mutant, expression of senescence transcription factors (*OsWRKY23* and *OsWRKY72*) and senescence-associated genes (*Osl2, Osl30, Osl43, Osl85, Osh36*, and *Osh69*) were examined using real-time PCR (Figures 3C,D). All eight genes, particularly *Osl43* and *Osl85*, exhibited elevated expression in the leaf sheath of the *sles* mutant, compared to wild type, consistent with the early senescence phenotype.

### HR-like lesions in the *sles* mutant

**Figure 4:**
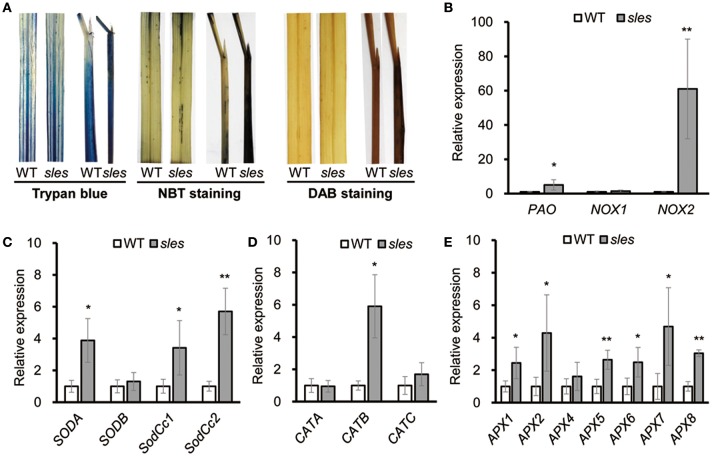
ROS accumulation in the *sles* mutant leaf sheath. **(A)** Trypan blue, NBT and DAB staining of penultimate leaves and leaf sheaths in wild-type and *sles* mutant plants after heading. **(B)** Expression of genes encoding ROS-generating enzymes in wild-type and *sles* mutant **(C–E)** Expression levels of ROS detoxification-related genes in wild-type and *sles* mutant. Real-time PCR (three biological replicates and three technical replicates) was performed with leaf sheath samples from wild-type and from areas with legion mimic spots in the *sles* mutant. Asterisks indicate the statistical significance level according to Student's *t*-test: ^**^P < 0.01 and ^*^P < 0.05.

Histochemical markers were examined to investigate putative mechanisms underlying the development of lesion mimic spots in the *sles* mutant (Figure 4A). The leaf sheath of *sles* mutant exhibited strong blue color of cells compared to that of wild type after staining with trypan blue, which is a histochemical indicator of irreversible membrane damage or cell death. There was no evidence of ROS production in the wild-type leaf sheath, but the pattern of NBT staining, an indicator of O${}_{2}^{-}$ accumulation, correlated strongly with lesion formation on the *sles* mutant leaf sheath. In leaves, there were negligible differences in ROS production between *sles* mutant and wild type. Similar results were obtained with 3,3′-diaminobenzidine (DAB) staining, which indicated H_2_O_2_ accumulation. These results confirmed that ROS accumulation in the leaf sheath of *sles* mutant lead to cell death and ultimately accelerated senescence.

### ROS homeostasis-related gene expression in the *sles* mutant

NADPH oxidase (NOX) and polyamine oxidase (PAO) are major ROS sources. Expression of *NOX1, NOX2*, and *PAO* was significantly increased in the spotted region of *sles* mutant leaf sheath (Figure 4B). Complex antioxidant systems in diverse subcellular compartments tightly regulate the abundance of intercellular ROS. These ROS scavenging systems include major enzymes, such as superoxide dismutase (SOD), catalase (CAT), and ascorbate peroxidase (APX), that coordinately function in ROS detoxification (Mittler et al., 2004). As *sles* mutants exhibited enhanced ROS accumulation, we next examined gene expression of ROS scavenging genes (*SODA, SODB, SodCc1, SodCc2, CATA, CATB, CATC, APX1, APX2, APX3, APX4, APX5, APX6, APX7*, and *APX8*) (Figures 4C–E) and found that most were significantly upregulated in the *sles* mutant compared to wild type.

### Blast resistance in the *sles* mutant

**Figure 5:**
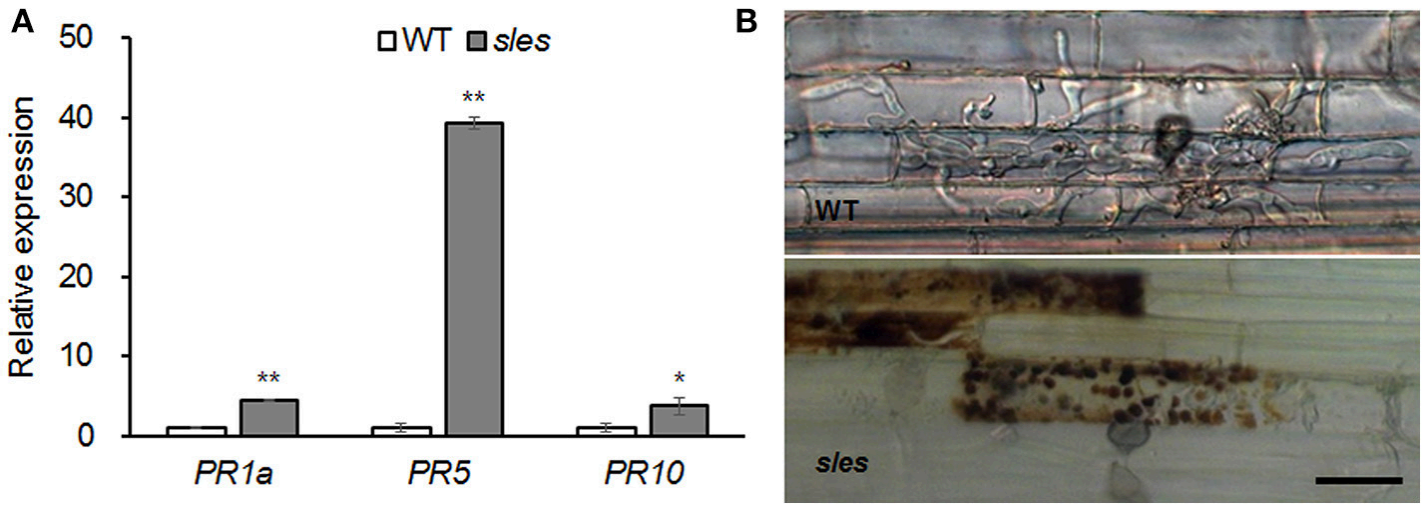
Blast resistance in the *sles* mutant leaf sheath. **(A)** Expression of pathogenesis-related marker genes. Real-time PCR was performed with leaf sheath samples from wild-type and from areas with legion mimic spots in the *sles* mutant. Asterisks indicate the statistical significance level according to Student's *t*-test: ^**^P < 0.01 and ^*^P < 0.05. **(B)** The excised leaf sheath from 50-day-old rice seedlings of WT and *sles* mutant was inoculated with conidial suspension (1 × 10^4^ conidia/ml). Samples were harvested and observed 48 h after inoculation. Three biological replicates and three technical replicates were performed for each experiment. Bar = 25 μm.

ROS contribute to accelerated transcriptional activation of *PR* genes, leading to production of antimicrobial secondary metabolites and localized cell death (Zurbriggen et al., 2010). As ROS accumulation was observed in the spotted region of the *sles* mutant leaf sheath, expression of three *PR* marker genes (*PR1a, PR5*, and *PR10*) associated with the defense response was examined (Figure 5A). All the *PR* genes were significantly upregulated in the *sles* mutant compared to wild type.

To evaluate response of the *sles* mutant to rice blast, the development of infectious hyphae (IH) within the host cells was observed using an excised leaf sheath assay (Figure 5B). IH actively grew and occupied 5–7 cells neighboring the primary infected cells by 48 h after inoculation in wild type. However, IH were mostly restricted to the primary infected cell, and there was an abundant accumulation of dark brown granules along IH in *sles* mutant. These results indicated that *sles* mutant conferred significantly enhanced resistance to rice blast compared to wild type.

### Genetic analysis of the *sles* mutant

F_1_ and F_2_ plants from crosses between the *sles* mutant and M.23 were used to determine whether the phenotype was dominant or recessive, and whether the *sles* mutant phenotype was controlled by multiple genes or by a single gene. F_1_ plants exhibited the wild-type phenotype, indicating that the *sles* mutant phenotype was recessive. The F_2_ population contained 492 wild-type plants and 136 plants with the *sles* phenotype, fitting a 3:1 ratio [χ${}_{\left(3:1\right)}^{2}$ = 3.75 < χ${}_{\left(0.05\right)}^{2}$ = 3.84, *P* = 0.06]. In another population of 55 F_2_ individuals derived from *sles* mutant/Koshihikari, the phenotype of 46 plants were wild type and 9 plants were *sles* mutant phenotype, matching a 3:1 ratio [χ${}_{\left(3:1\right)}^{2}$ = 2.19 < χ${}_{\left(0.05\right)}^{2}$ = 3.84, *P* = 0.14]. This indicated that the *sles* phenotype was controlled by a single recessive nuclear gene.

### Genetic mapping and identification of the *SLES* gene

**Figure 6:**
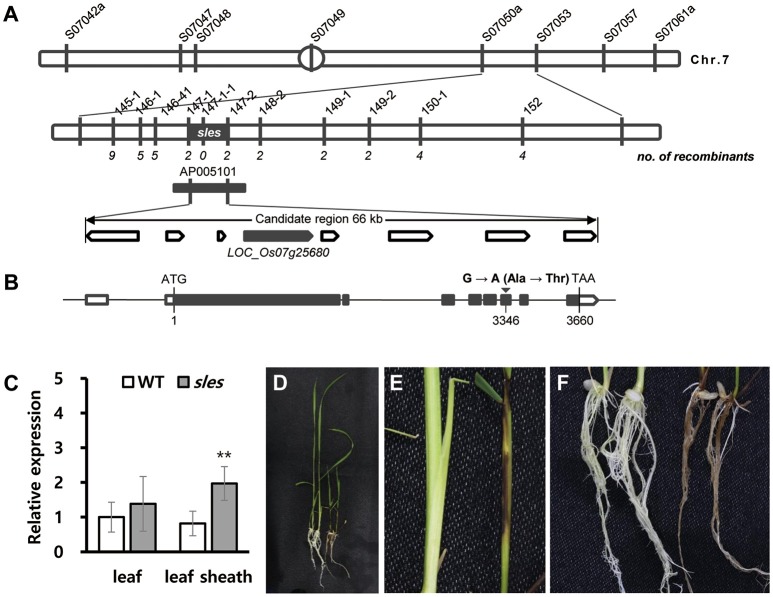
Fine-mapping and identification of *SLES*. **(A)** Fine-mapping of *SLES*. The *sles* locus was mapped to a 66 kb region on chromosome 7. **(B)** Schematic diagram of *SLES*. Black rectangles represent exons and the black inverted triangle represents the mutation site. **(C)** Expression of *SLES* gene on leaf and leaf sheath. **(D–F)** Phenotypic comparison of wild-type Dongjinbyeo (left) and the homozygous T-DNA insertion line (right). **(D)** Seedling. **(E)** Leaf sheath. **(F)** Root. Real-time PCR (three biological replicates and three technical replicates) was performed. Asterisks indicate the statistical significance levels according to Student's *t*-test: ^**^P < 0.01 and ^*^P < 0.05.

An F_2_ population derived from a cross between the *sles* mutant and M.23 was used to map the locus responsible for the *sles* mutant phenotype. BSA using 60 polymorphic STS markers evenly distributed across the 12 rice chromosomes was used for preliminary genetic mapping. BSA mapped the *sles* locus to the interval between STS markers S07050a and S07053 (Figure 6A). Using 628 F_2_ individuals with newly designed STS markers between the two flanking markers, the *sles* locus was mapped to the interval between markers 147-1 and 147-2, an ~66 kb physical distance in Nipponbare (Figure 6A). Eight predicted candidate genes were located within the 66 kb candidate region, and the region was encompassed by BAC clone AP005101 (Supplementary Table 2).

Sequence comparisons of candidate genes between wild type and the *sles* mutant revealed a single point mutation in the 6th exon of the *LOC_Os07g25680* candidate gene. Guanine (G) in the wild-type gene was changed to adenine (A) in the *sles* mutant gene, resulting in a single amino acid change from alanine to threonine at position 3,346 (Figure 6B). For coding regions, no other DNA sequence differences were detected in any other candidate genes. To verify the SNP, dCAPS markers were designed and used to screen the F_2_ mapping population. Primers used for PCR are listed in Supplementary Table 1. The genotype exhibited complete co-segregation with the matching phenotypes (Supplementary Figure 1A). To examine whether the SNP was present as a natural variant in other cultivars, dCAPS analysis of eight *japonica* and five *indica* rice cultivars was performed. None of the 13 cultivars exhibited an additional restriction fragment (Supplementary Figure 1B).

To understand the possible role of *SLES* in premature senescence with lesion mimic spots, we examined the transcriptional level of *SLES* during the formation of lesion mimic spots on the leaf sheath in *sles* mutant. The results showed that the *SLES* exhibited significantly elevated expression in the leaf sheath of the *sles* mutant compared to wild type (Figure 6C). However, there was no remarkable difference of the expression level in the leaf between wild type and *sles* mutant (Figure 6C).

### Validation of the mutation causing *sles* mutant phenotype

A T-DNA insertion line (3A-11526.R) from the Crop Biotech Institute, Department of Plant Systems Biotech, Kyung Hee University, was used to confirm that a single functional base substitution in *SLES* gene was responsible for the abnormal phenotype of *sles* mutants. This line has a T-DNA inserted into the first intron of LOC_Os07g25680 (Supplementary Figure 2A), which was confirmed by PCR analysis. Seven homozygous and five heterozygous T-DNA tagging mutants were identified (Supplementary Figure 2B). As with the *sles* mutant, even severer, lesion mimic spots appeared on leaf sheaths and roots in the homozygous T-DNA insertion lines, and seedling height was shorter than the wild type (the cultivar Dongjin) (Figures 6D–F). Homozygous T-DNA insertion lines exhibited weak growth vigor compared to wild type and eventually died within 4 weeks after germination. These results confirmed that the mutation in *SLES* was responsible for the *sles* mutant phenotype.

### SLES protein structure prediction

**Figure 7:**
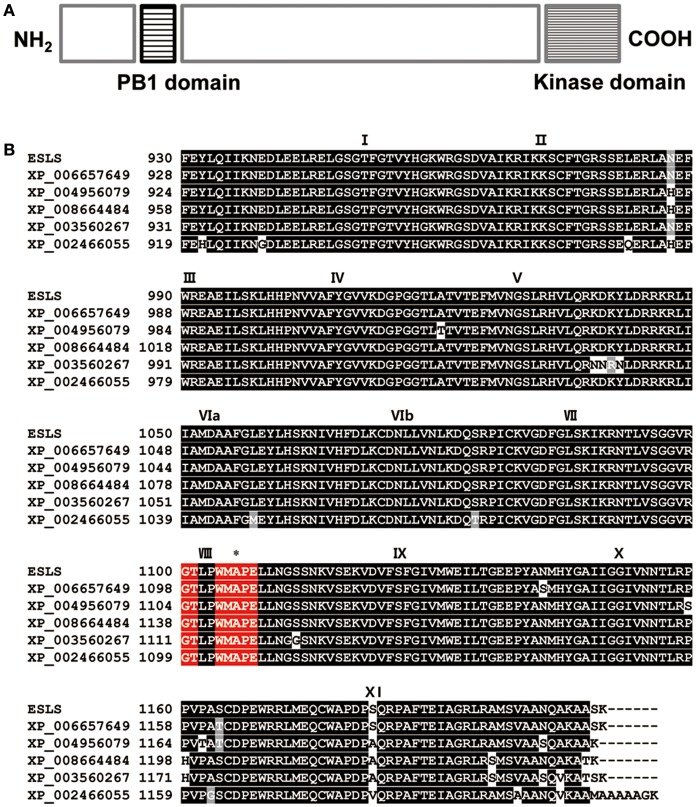
Protein sequence analysis of SLES. **(A)** Predicted schematic of the SLES protein. **(B)** Amino acid sequence alignment of the SLES kinase domain with that of other proteins indicated that SLES had a highly conserved kinase domain. Black boxes indicate identical residues and gray boxes indicate conservative substitutions. Roman numerals indicate the 11 characteristic sub-domains of protein kinases. The Raf specific motif is shown in red boxes. Asterisk indicates the position where a single amino acid change occurred in the *sles* mutant. Shown are: *Oryza brachyantha* (XP_006657649), *Setaria italic* (XP_004956079), *Zea mays* (XP_008664484), *Brachypodium distachyon* (XP_003560267), and *Sorghum bicolor* (XP_002466055).

Examination of the rice genome database revealed that the coding sequence (CDS) of *SLES* consisted of 3,660 nucleotides over 8 exons, and encoded a putative 1,219-amino acid protein. SLES contained Phox and Bem1p (PB1) domain at the N terminus and KD at the C terminus (Figure 7A). The SLES KD contained all 11 subdomains common to known protein kinases (Hanks and Quinn, 1991; Figure 7B). Bioinformatic analysis and multiple amino acid sequence alignment of the predicted KD indicated a conserved catalytic and RAF-specific signature GTXX (W/Y) MAPE, which classified SLES as a Raf MAPKKK (Figure 7B; Rao et al., 2010). SLES homologs were identified within monocots such as *Oryza brachyantha, Setaria italica, Brachypodium distachyon, Sorghum bicolor*, and *Zea mays* with 60–92% amino acid identity. However, no clear co-orthologues were identified in eudicots. None of these predicted proteins have been characterized to date.

## Discussion

Several rice mutants associated with lesion mimic spots that result in early senescence, such as *spl5, lmes1*, and *lmes2*, have been identified (Chen et al., 2012; Li et al., 2014; Xing et al., 2016). However, to the best of our knowledge, no LMMs in which lesion mimic spots are found on the leaf sheath have been identified in rice to date. The *sles* mutant identified in this study exhibited lesion mimic spots on the leaf sheath. Further analysis revealed that these lesion mimic spots were attributable to ROS accumulation.

In the *sles* mutant, total chlorophyll content and Chla and Chlb levels were significantly lower in the spotted region than in wild type, whereas chlorophyll levels in non-spotted regions in the *sles* mutant did not differ from wild type. The ratios of Chla/Chlb in the non-spotted regions and in wild type were in the 2.5–4.0 range. The Chla/Chlb ratio in the *sles* mutant spotted regions was significantly lower than that in wild type, indicating that Chla levels in the mutant were relatively more diminished than Chlb levels. *SGR, NYC1, NYC3*, and *NOL* play important roles in chlorophyll degradation. Overexpression of *SGR* and *NYC3* accelerated chlorophyll degradation in developing leaves. Moreover, Chlb content was slightly lowered at the late stage of senescence in *nol-1, nyc1-2*, and *nol-1 nyc1-2* mutants (Park et al., 2007; Sato et al., 2009; Wei et al., 2013). In the *sles* mutant, expression of chlorophyll degradation genes, particularly *SGR*, was markedly higher than in wild type. These results suggest that chlorophyll content in the *sles* mutant was reduced by activation of chlorophyll degradation genes resulting in senescence of the leaf sheath. Leaf senescence is mediated by a large number of genes, such as senescence transcription factors (*WRKY23* and *WRKY72*) and *SAGs* (*Osl2, Osl30, Osl43, Osl85, Osh36*, and *Osh69*) (Lee et al., 2001; Zhou et al., 2013). Expression of *WRKYs* and *SAGs*, particularly *Osl43* (stress response) and *Osl85* (fatty acid metabolism), was significantly increased in the leaf sheath of the *sles* mutant compared to wild type. Moreover, a large amount of irreversible membrane damage or cell death were observed in the leaf sheath of *sles* mutant. Taken together, the phenotypic, physiological, biochemical and molecular observations indicate that early senescence occurs in the leaf sheath of the *sles* mutant.

The results outlined above showed that HR-like cell death, leading to early senescence, occurred in the *sles* mutant; however, the cause of the lesion mimic spots on the leaf sheath remained unclear. Substantial ROS accumulation [superoxide (O${}_{2}^{-}$) and hydrogen peroxide (H_2_O_2_)] was detected in leaf sheath of the *sles* mutant. NADPH oxidase (NOX) and polyamine oxidase (PAO) are the main ROS sources (Langebartels et al., 2002). An *OsSRFP1* overexpression line with enhanced levels of *NOX* showed high levels of ROS accumulation (Fang et al., 2015). Overexpression of *AtPAO3* also resulted in increased production of ROS (Sagor et al., 2016). Expression of *NOX2* and *PAO* was substantially elevated in the *spotted leaf* sheath of the *sles* mutant compared to wild type. These results reveal that the elevated expression of genes encoding ROS-generating enzymes may have led to ROS accumulation in spotted regions of the *sles* mutant leaf sheath. As a large amounts of hydrogen peroxide and superoxide anion accumulated in the spotted region of the *sles* mutant leaf sheath, the expression of gene encoding scavenging enzymes, especially those located in the chloroplast, such as *SodCc1, SodCc2, APX5, APX6, APX7*, and *APX8*, was significantly higher than in wild type.

ROS accumulation not only triggers senescence but also activates the expression of defense genes such as *PR* genes (Dangl and Jones, 2001; Zentgraf and Hemleben, 2008). Overexpression of these *PR* genes may enhance plant tolerance to pathogen infections. For instance, overexpression of *OsPR1* in tobacco showed enhanced host tolerance to *Phytophthora nicotianae, Palstonia solanacearum*, and *Pseudomonas syringae* (Sarowar et al., 2005); overexpression of *JIOsPR10* in rice enhanced tolerance to *Magnaporthe oryzae* (Wu et al., 2016); and overexpression of *PR5* in rice enhanced tolerance to *Rhizoctonia solani* (Datta et al., 1999). Expression of *PR* genes, especially *PR5*, was significantly higher in the leaf sheath of the *sles* mutant than in wild type and showed significantly enhanced disease resistance to *M. oryzae* by restricting the development of infectious hyphae.

A database search and sequence analysis suggested that SLES contained a conserved protein KD and was a member of the Raf MAPKKK family. Although MAPK cascades have been identified and characterized in rice, little is known about members of the MAPKKK gene family and their functions and regulation in rice. MAPKKKs act upstream of MAPK cascade composed of three classes of enzymes: MAPKKK, MAPKK, and MAPK. Upstream signals activate MAPKKKs, which then phosphorylate MAPKKs. MAPKKs in turn activate a specific MAPK. The downstream targets of MAPKs can be transcription factors, phospholipases, or cytoskeletal proteins (Sturgill and Ray, 1986; Lin et al., 1993; Tian et al., 2017). In general, substrates of kinase are found at relatively constant level in most tissues. However, Gould et al. (1984) revealed that some kinase substrates are only expressed at high level in certain tissues. Since sles protein loses the normal function of kinase activity, we suggest that a specific protein kinase substrate regulating ROS homeostasis specifically in leaf sheath might not regularly conduct its normal function, resulting in ROS accumulation in the leaf sheath of *sles* mutant. However, further experiments are necessary.

Several studies reveal that MAPKKK gene family is involved in plant defense/stress responses as well as ROS homeostasis regulation. Studies of *MEKK1*, the MAPKKK of the flagellin cascade, revealed that MEKK1 conferred resistance to both bacterial and fungal pathogens (Asai et al., 2002). Overexpression of *TaFLR* (a wheat MAPKKK gene) activated *PR* genes, such as *PR2a* and *PR3*, and resulted in increased resistance to *Fusarium graminearum* (Gao et al., 2016). In the *sles* mutant, markedly increased ROS accumulation was observed in accordance with induced expression of genes encoding ROS generating enzymes. Moreover, pathogenesis-related genes, especially *PR5*, were activated and pathogen resistance was enhanced in the *sles* mutant compared to wild type. Taken together, we suggest that SLES might suppress production of ROS associated with pathogen defense mechanism, thus leading to HR-like cell death on the leaf sheath and prevents the further pathogen infection of the *sles* mutant.

In this study, the *SLES* gene was characterized and isolated. Further examination of *SLES* will facilitate a better understanding of the molecular mechanisms involved in ROS homeostasis and may also provide opportunities to improve pathogen resistance in rice. Furthermore, as *SLES* is a Raf MAPKKK, the *sles* mutant is ideal for studies of MAPK cascades.

## Author contributions

DL performed research, analyzed data, and wrote the manuscript. GL, BK, YY, and JS performed research and analyzed data. SJ and YL phenotyped samples and analyzed data. Y-HL and SeK performed blast resistance evaluation and analyzed data. JL designed research and performed research. SuK and H-JK designed research and wrote the manuscript.

### Conflict of interest statement

The authors declare that the research was conducted in the absence of any commercial or financial relationships that could be construed as a potential conflict of interest.
