# Supplementary material for: Identification of a Spotted Leaf Sheath Gene Involved in Early Senescence and Defense Response in Rice
Source: Front Plant Sci. 2018 Sep 5;9:1274. doi: 10.3389/fpls.2018.01274 (PMC6134203; doi:10.3389/fpls.2018.01274)
Supplement: Supplementary file 1 [file Presentation_1.PDF]

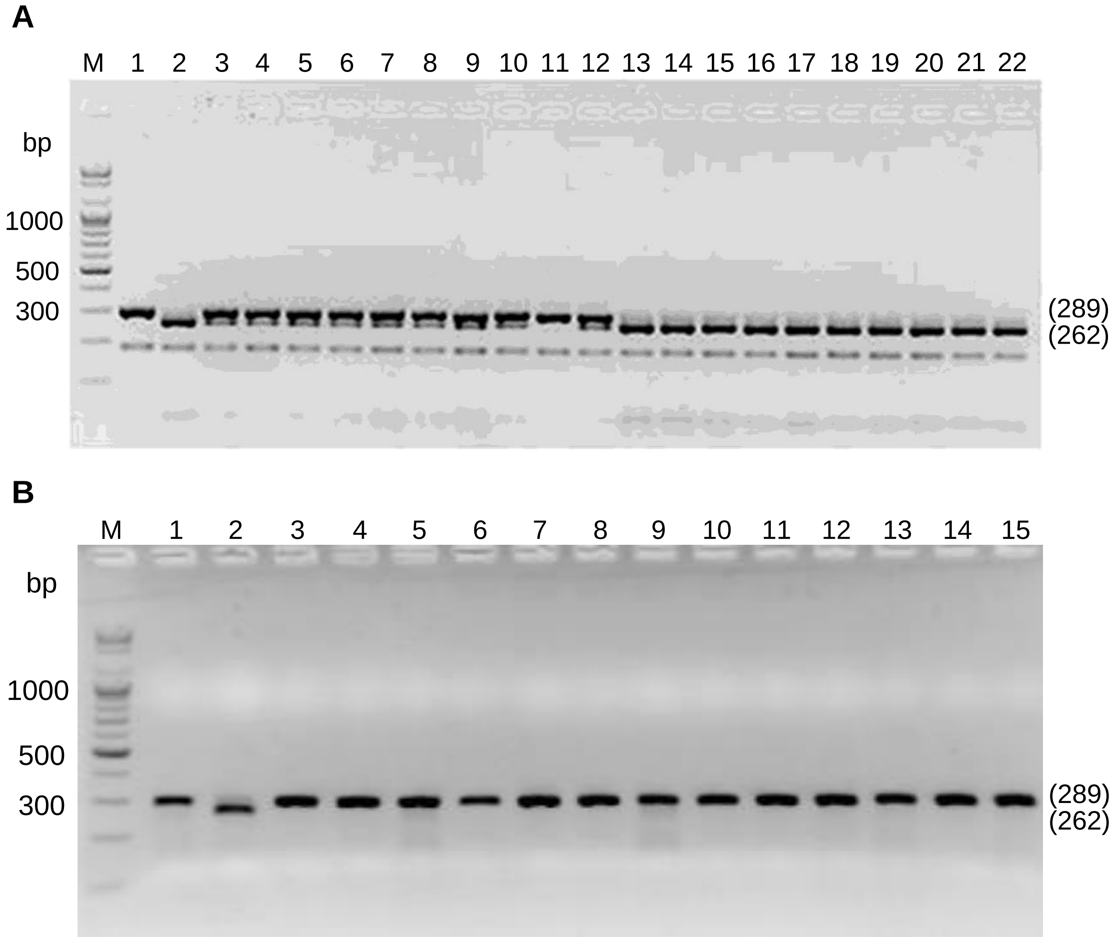

Fig. S1 dCAPS maker analysis. **(A)** Co-segregation with the *sles* mutant phenotype was analyzed by size comparison of PCR products from the F_2 population. Lanes 1-2, WT and *sles* mutant, respectively; lanes 3-12, normal homozygotes and heterozygotes; 13-22, *sles* mutant homozygotes. **(B)** Confirmation of the splice variation in *SLES* gene by analysing PCR product size in *sles* mutant and 14 rice varieties. Lanes 1-2, WT and *sles* mutant, respectively; lanes 3-10 *japonica* rice varieties (Hwachong, Dongjin, Nipponbare, Ilpum, Hapcheon, Kunmingxiaobaigu, Dainxi4, Tong88-7) and lanes 11-15 *indica* rice varieties (M.23, Dasan, IR64, Unkwang, Giza178).

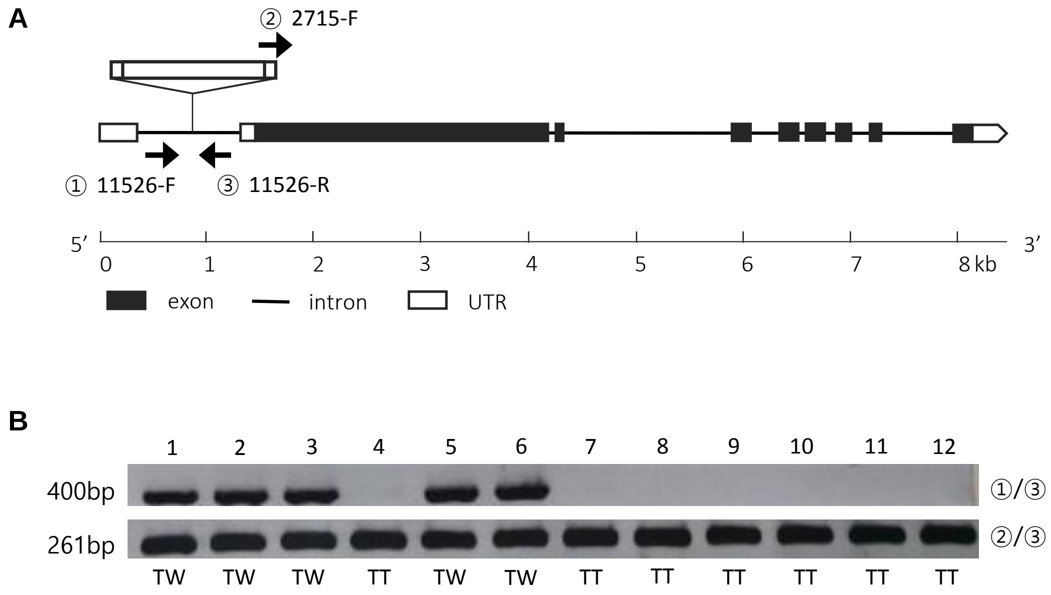

Fig. S2 Analysis of the *SLES* locus in the T-DNA insertion line. **(A)** Schematic diagram of the T-DNA insertion. *Arrows* indicate primers. **(B)** Genotype of the T_1 plants evaluated by the PCR amplification with primer combinations of 11526-F/11526-R and 2715-F/11526-R. *TT* homozygous mutant, *TW* heterozygous.

Table S1 Primers used in this study

Purpose	Primer name	Forward primer (5' → 3')	Reverse primer (5' → 3')
Mapping	S07050a	CTCCACTTATGGCAGCGAAT	CAAGTGAAGTGGGAGCAGGT
	S07053	CGAAACTTTGGGACGAAATG	CGTCCACCATTCACTGTCAC
	147-1	GCCAGTACATCCTCGTTCGT	GAGCATGGAGGATCCAAAGA
	147-1-1	AACCATGCATTGCAACAGAC	GCTGAATCAAACAGGGCTTT
	147-2	CTTGTGCGCCTGGAATATG	TCGATAAACTCATCCGACGTA
Real-time PCR	SGR	GGCCTCCGCTACTACATCTT	GGGAGGTTGGAGTGGAAGTA
	NYC3	TGTGCTCCAAAGGGACAAAT	GATTCTGGCACCTGCTGTTT
	NYC1	GCTTGCCTTGGTTACTGCAT	TTCAGCCCAATTACGGATTC
	NOL	ACATCCGTTTTCTCACAGGC	GGCAGCAAAACGGACACTAA
	WRKY23	TACCGATGGAGGAAGTACGG	CTTGCTTCTTCACGTTGCAC
	WRKY72	GCTTCCCACATGTTCCAGTAG	TTTTCCTGCTAACATCCATCG
	Osl2	GCAGACAACAAATCGCCAAAT	TCTCCAGCAACTCTAACCAGCAT
	Osl30	GAGAAATCCCTTGAAGCCAA	CACAAAGCAGTGAAAGCACA
	Osl43	TGTGACAAGTGCTAATAATACATACGA	CCAGACCTTCCAAAGAATCCAAC
	Osl85	TCCAGGATGTGATGAGGATTATTC	GCGTGCTGTAGTTCAGTCTGTAAAG
	Osh36	GTGCACCATGCACTTAATCC	CACCGACCCTTCCTGTAGTT
	Osh69	ACGAGCTACACGCCTACCTT	ACTTCCTTGCCAGAAGCACT
	PAO	CCTAGCCAAGAAGTGTTGCC	TCGCTCCCATGAAGACCTTT
	NOX1	AGGCCGACTGCTTCCTCT	CACTGACAATTGCAGCAGGT
	NOX2	ACTGCTTCCTCTTCGCCTCT	CTCTGTCAGCCCAGCAGTT
	SODA	ATCTGGATGGGTGTGGCTAGCTTT	AGTACGCATGCTCCCAGACATCAA
	SODB	TCCGCCGTATAAACTTGATGCCCT	TGGGTTGCCGTTGTTGTATGCTTC
	SodCc1	GTGCATGCCGATCCTGATG	CTGGGAGATGGAAGGTGAGT
	SodCc2	TGTGACGGGAAGTGTCTCTG	AGTAAGGGGGATCTGGCTGT
	CATA	CAACCGCAACGTCGACAACTTCTT	TTCACCGGCAGCATCAGGTAGTTT
	CATB	GCTTGCTTTCTGCCCAGCGATAAT	AAATAGTTTGGGCCAAGACGGTGC
	CATC	CCACGAGATCAGGAGCATCT	TCCGTGACTGAAGCAGATTG
	APX1	AGGTGCCACAAGGAAAGATCTGGT	TCAGCAGGGCTTTGTCACTAGGAA
	APX2	TGGGAAGATGCCACAAGGAGAGAT	TCCGCAGCATATTTCTCCACCAGT
	APX4	GAAGCTCCCAACTGACAAGG	TTGTGCGATTCAGCGTAGTC
	APX5	CTGAAGCTCATGCCAAACTG	TATCACTCGCCAAAATGCAG
	APX6	CTGAAGCTCATGCCAAACTG	CTTGTTCATTCGCCAAAATG
	APX7	TACGCAGAGGACCAAGAAGC	ACTTCAGCGATCTGGCTCAT
	APX8	CAGTAACGGTAGGAGCAGCA	TACTCCGCCCTGATCTTCTG
	PR1a	TTCATCACCTGCAACTACTCG	TGCATAAACACGTAGCATAGCAT
	PR5	ATCGACGGCTACAACGTC	GTGTCTTGGTGTTGTCTTCG
	PR10	CACCATCTACACCATGAAGC	AGCACATCCGACTTTAGGAC
	eEF1a	AGCACGCTCTTCTTGCTTTC	TGTAGCCGACCTTCTTCAGG
dCAPS	HphI	GGGACTCTCCCATGGGTG	GCCAAAGGAAAATACATCAACC
T-DNA insertion	11526	CGATCGGGATTGTTAGCTGT	TTCAGCAACACGTACTAAAATGA
	2715	AGCACCCCAAGTTAGTCATGT	

Table S2 Predicted genes in the mapped *sles* region (66 kb)

**Gene name**	**Size (bp)**	**Predicted function**
LOC_Os07g25650	1890	Predicted expressed protein
LOC_Os07g25660	345	Predicted expressed protein
LOC_Os07g25670	726	Predicted expressed protein
LOC_Os07g25680	3660	Protein kinase domain containing protein
LOC_Os07g25690	354	Subtilisin N-terminal region family protein
LOC_Os07g25700	591	Predicted expressed protein
LOC_Os07g25710	1281	myb-like DNA-binding domain containing protein
LOC_Os07g25730	213	Predicted expressed protein
